# The Association Between Dietary Protein Intake and Gallstone Disease: A Case–Control Study

**DOI:** 10.1155/grp/7362453

**Published:** 2025-11-20

**Authors:** Amir Sadeghi, Nastaran Raissi-Dehkordi, Azita Hekmatdoost, Moloud Ghorbani, Reyhaneh Rastegar, Mohammad Reza Zali, Negar Raissi-Dehkordi, Zahra Yari

**Affiliations:** ^1^Gastroenterology and Liver Disease Research Center, Research Institute for Gastroenterology and Liver Disease, Shahid Beheshti University of Medical Science, Tehran, Iran; ^2^Clinical Nutrition and Dietetics Department, Faculty of Nutrition Sciences and Food Technology, National Nutrition and Food Technology Research Institute, Shahid Beheshti University of Medical Sciences, Tehran, Iran; ^3^Department of Nutrition, Faculty of Medicine, Mashhad University of Medical Sciences, Mashhad, Iran; ^4^Department of Nutrition Research, National Nutrition and Food Technology Research Institute and Faculty of Nutrition Sciences and Food Technology, Shahid Beheshti University of Medical Sciences, Tehran, Iran

**Keywords:** animal protein, dairy protein, gallstone, protein

## Abstract

**Background:**

Diet is a modifiable risk factor for gallstone formation and influence the risk of gallstone disease (GSD). The present study was designed with the aim of investigating the association between different dietary protein and the risk of GSD.

**Methods:**

This case–control study was conducted on 189 patients diagnosed with GSD and 342 controls. Intake of total protein and its subgroups was measured based on food frequency questionnaire. Using multiple logistic regression models, crude and multivariable adjusted odds ratios (ORs) and 95% confidence intervals (CIs) were estimated.

**Results:**

Total protein intake can have protective effects against GSD and its beneficial effect was more in moderate consumption (OR_T2 vs.T1_ = 0.2, 95% CI 0.1–0.4) than high consumption (OR_T3 vs.T1_ = 0.39, 95% CI 0.23–0.66) (*p* for trend < 0.001). The relationship between dairy protein intake and the risk of GSD was U-shaped, so that moderate intake was associated with a reduced risk (OR_T2 vs.T1_ = 0.76) and higher intake was associated with an increased risk of the disease (OR_T3 vs.T1_ = 1.5) (*p* for trend = 0.027). Vegetable protein as a protective factor and animal protein as a risk factor showed a significant relationship with the risk of GSD.

**Conclusion:**

Dietary protein intake, especially vegetable protein, may protect against GSD, while animal protein may be a predisposing factor. Dairy protein protects against GSD when consumed in moderation, but higher intakes may increase the risk of GSD.

## 1. Introduction

Gallstones are pathological masses formed due to excessive levels of cholesterol or bilirubin in the gallbladder. Prevalence of gallstone disease (GSD) is approximately 10–20% worldwide [1], of which more than 20% eventually become symptomatic. Gallbladder stones are mostly made of cholesterol (> 90%), while a small percentage are black and brown pigment stones [2]. Pain in the right upper quadrant of the abdomen is the classic presenting symptom, which often occurs following intake of greasy or spicy food and may be accompanied by nausea and vomiting [3]. Several modifiable and nonmodifiable risk factors have been identified for GSD; the main nonmodifiable risk factors are aging, female gender, ethnicity, and genetic predisposition [4], while obesity, dyslipidemia, hyperinsulinemia, physical inactivity, rapid weight loss, and dietary factors are among the modifiable risk factors for GSD [5]. Dietary intakes recognized as GSD risk factors include high intake of calories or carbohydrates and low intake of fiber [1].

Dietary protein intake may influence the occurrence of GSD. A number of animal studies have demonstrated a protective effect for vegetable proteins against GSD through reduced biliary cholesterol, in addition to decreased amounts of lithogenicity and crystallization [6]. Despite the positive results from animal studies [6], human studies [7, 8] on the role of proteins are lacking and present conflicting results; a large prospective cohort study on US women found vegetable protein to protect against GSD occurrence, as demonstrated by the decreased number of cholecystectomies performed during the 20-year follow-up period. No association was found between animal protein intake and cholecystectomies [7]. Similar results were reported from a study on postmenopausal women, in whom total protein and vegetable protein reduced the risk of GSD, while animal protein did not alter the occurrence of gallbladder stones [8]. In contrast, two case–control studies found no relationship between protein consumption and GSD [9, 10]. A large epidemiologic survey on an Italian population (*n* = 29,584, GSD = 1801) found proteins to be protective against GSD, but only in the male participants [11].

In the presenting study, we evaluated the role of dietary proteins, including total, dairy, vegetable and animal proteins, in the development of GSD. This study could provide insight for researchers regarding the involvement of proteins in the odds of GSD development.

## 2. Methods and Materials

### 2.1. Patients Selection

**Figure 1:**
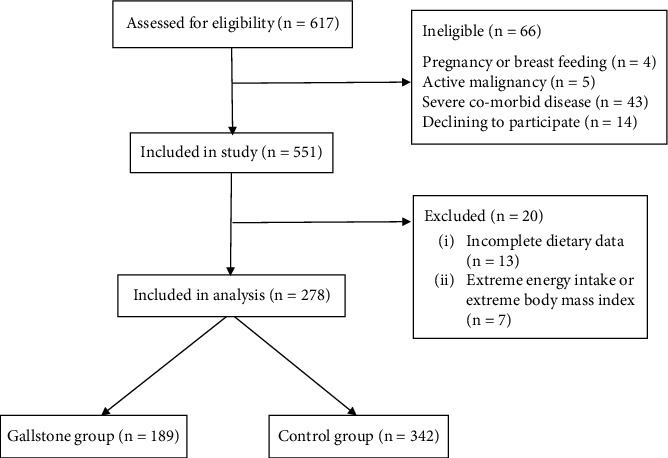
Flowchart of study enrollment.

We conducted this case–control study in the inpatient clinic of the Research Institute of Gastroenterology and Liver Diseases, Taleghani Hospital, Tehran, Iran. The detailed methodology of this study has been previously described [12]. Briefly, cases were defined as adult patients diagnosed with gallstone disease (GSD) within the preceding month and willing to participate. Controls were selected from other hospital departments (e.g., orthopedics, biochemistry) and had no history of GSD or other gastrointestinal disorders. Eligibility criteria for both groups included age ≥ 18 years, absence of diabetes mellitus or cardiovascular disease, and no adherence to special diets or use of medications affecting lipid or glucose metabolism (e.g., ursodeoxycholic acid, weight loss agents). Individuals who were pregnant or lactating, or had a history of autoimmune, inflammatory, infectious, or malignant diseases were excluded. A detailed flowchart of participant recruitment is presented in Figure 1.

The study protocol was approved by the Research Institute of Gastroenterology and Liver Diseases Ethics Committee (ID: IR.SBMU.RIGLD.REC.1396.159). The study was conducted in accordance with the latest version of Declaration of Helsinki. Written informed consent was obtained from all included cases and controls.

### 2.2. Dietary Intake Assessment

A 168-item semi-quantitative Food Frequency Questionnaire (FFQ) was used to explore the dietary habits of patients [13]. Nutritionist IV software was used to analyze the assembled data. Energy and nutrient content of participants' diet was calculated using the food composition table of the United States Department of Agriculture. Total protein and the intakes of dairy, vegetable (grain, legumes, and nuts), and animal (red meat, poultry, eggs, processed meat, and fish) protein were calculated and reported in grams per day. Since newly diagnosed patients were included in the study, the likelihood of dietary changes after diagnosis was minimized, and therefore, dietary intake assessment was performed before any disease-induced dietary changes.

### 2.3. Data Collection

In-person interviews were conducted by a trained interviewer to collect information regarding demographics, weight and height measurements, any relevant medical history, and tobacco use and alcohol consumption. A digital scale was used to measure body weight which was then rounded to the nearest 100 g [14]. A portable nonstretch meter was used to measure height and rounded to the nearest 0.5 cm. The International Physical Activity Questionnaire (IPAQ) was used to assess physical activity [15].

### 2.4. Statistical Analysis

Kolmogorov–Smirnov test and histogram charts were used to test for normality, which revealed that all quantitative data followed a normal distribution. Analysis of variance (ANOVA) test was used for quantitative data and the chi-square test was used for qualitative variables. Participants were separated into three groups (tertiles) based on total protein intake, in addition to intake of vegetable, animal, and dairy protein. Participants were categorized into three groups based on 33^rd^ and 66^th^ percentile values for protein intake to best fit the data distribution, simplify interpretation, and perform a comparison between triplicates without extreme differences in sample size between groups. Risk of GSD in relation to total and subtypes of dietary protein intake was calculated using logistic regression models. We adjusted for potentially confounding factors including age, sex, energy intake, body mass index (BMI), physical activity, and tobacco and alcohol consumption. Risk of GSD in each tertile was reported using odds ratio (OR) and 95% confidence interval (CI) while considering related risk factors. All statistical analyses were performed using SPSS version 19.0 (SPSS Inc., Chicago, United States). *p* Value < 0.05 was the threshold of significance.

## 3. Results

Five hundred thirty-one patients including 189 cases and 342 controls were included in the present study (202 males and 329 females). A comparison of the characteristics of the participants can be seen in a previously published article from the same project [12].  Table 1 describes the baseline general characteristics of patients. Mean age of participants was 52.9 ± 13.3 (youngest: 21–oldest: 91). Based on total dietary protein intake, participants were divided into three groups. The three groups were similar regarding age, alcohol consumption, physical activity, and BMI, but men, smokers, and alcohol drinkers were more likely to consume more total protein, while GSD patients were more likely to consume less protein overall. Overall, mean daily calorie intake was 2364.5 ± 627.2 kcal and mean BMI was 26.96 ± 4.36 kg/m^2^.

Table 2 describes risk of gallbladder stones based on different types of dietary protein. Patients with GSD were more likely to consume significantly less total protein. Frequency of patients in first tertile of total protein was 78, second tertile was 52 and third tertile was 59 (*p* for trend 0.006). Also, by increasing the intake of vegetable protein, the number of patients decreased significantly (*p* for trend 0.045) and by increasing the intake of animal protein, the number of patients increased significantly (*p* for trend 0.029). There was no noticeable difference in the number of GSD cases based on tertiles of dairy protein intake.

The moderate intake of total protein (second tertile 64–82 g/day) decreased the risk of GSD by half (OR = 0.5, 95% CI 0.32–0.78), while more intake of total protein intake (third tertile) was associated with a 39% reduction in risk of GSD (OR = 0.61, 95% CI 0.395–0.93; *p* for trend = 0.006) in a crude model. Adjusting the results for age and gender eliminated the significance of this association, but further adjustment for other confounding variables, including energy intake, BMI, physical activity, smoking, and alcohol, considerably strengthened the initially observed patterns (OR_T2 vs.T1_ = 0.2, 95% CI 0.1–0.4, and OR_T3 vs.T1_ = 0.39, 95% CI 0.23–0.66; *p* for trend < 0.001). The relationship between dairy protein intake and the risk of GSD was U-shaped, so that its moderate intake was associated with a reduced risk (OR_T2 vs.T1_ = 0.76, 95% CI 0.45–1.21) and higher intake was associated with an increased risk of the disease (OR_T3 vs.T1_ = 1.5, 95% CI 0.8–1.89) (*p* for trend = 0.027). The results were the same in the crude and adjusted models.

Vegetable protein decreased the propensity for GSD, so higher amounts of vegetable protein consumption resulting in lower risk of GSD occurrence (OR_T2 vs.T1_ = 0.71, 95% CI 0.46–1.1, and OR_T3 vs.T1_ = 0.64, 95% CI 0.42–1; *p* for trend = 0.013). Adjustment for confounding variables yielded similar results.

**Figure 2:**
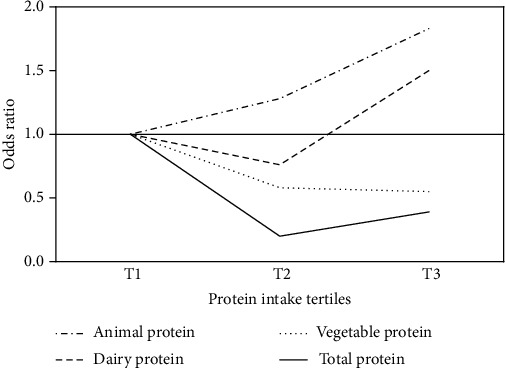
The association between different types of protein intake and gallstone with adjustment for age, sex, energy intake, BMI, physical activity, smoking, alcohol.

In contrast, animal proteins increased the risk of GSD, with higher intake of animal protein further increasing the risk of GSD (OR_T2 vs.T1_ = 1.1, 95% CI 0.7–1.56, and OR_T3 vs.T1_ = 1.34, 95% CI 0.6–2.3; *p* for trend = 0.06). Adjustment for confounders reinforced this association (OR_T2 vs.T1_ = 1.28, 95% CI 1.1–2.7, and OR_T3 vs.T1_ = 1.83, 95% CI 1.1–3.4; *p* for trend = 0.038). A visual summary of our findings can be found in Figure 2.

## 4. Discussion

The findings of our case–control study indicated that dietary protein intake, both total dietary protein and its subgroups, including vegetable, dairy, and animal protein, may be related with the risk of GSD. These findings suggested an inverse association between total and vegetable protein intake with GSD risk, and a positive association between animal protein intake and GSD risk. An interesting pattern was observed regarding total and dairy protein. A moderate intake of dairy protein appeared to be protective against GSD, while a higher intake increased the propensity for gallstone formation. Similarly, an inverse relationship between total protein intake and GSD was observed at baseline, but this trend appeared to be attenuated at higher total protein intakes. The greatest benefit against GSD was observed in moderate total protein intake rather than higher amounts. Studies examining vegetable versus animal protein sources often show more modest protective effects, suggesting that specific protein types may influence the magnitude of the association [8, 16]. However, a recent meta-analysis examining GSD risk and diet generally reinforces the protective role of a moderate protein intake, although the size of the effect varies depending on the study populations and methods [17].

The effect of adhering to a vegetarian diet in protecting against GSD has previously been studied, with conflicting results. These variations in the findings may stem from discrepancies in the definitions of animal protein sources or potential misclassification. A prospective cohort study on 4839 participants (GSD cases = 104) demonstrated that vegetarian diet decreased the risk of GSD occurrence in women by almost half, but did not show protective effects in men [18]. Other studies corroborate this protective role [19, 20], and a systematic-review and meta-analysis (GSD cases = 33,983) demonstrated that fruit and vegetable consumption decreased the risk of GSD incrementally [21]. In contrast, a large study on 49,652 British adults (cases = 1182) with a mean follow-up of 13.85 years, one-third of whom were vegetarians, found the vegetarian diet to modestly but significantly increase the risk of GSD [22]. However, a study on 2147 individuals using ultrasound examination to detect gallstones found no association between GSD and vegetarian diet (GSD cases = 171) [23]. Apart from studies on the vegetarian diet and general vegetable consumption, vegetable protein itself may reduce the occurrence of GSD. Strong evidence supports the protective role of vegetable protein against gallbladder stones in comparison to animal proteins [24, 25], and large, prospective cohorts support these findings; the largest prospective cohort on the link between protein consumption and GSD (the Women's Health Initiative), based on 130,859 postmenopausal women, found that vegetable protein to be modestly but significantly protective against GSD [8]. Similarly, vegetable protein was protective against GSD in The Nurses' Health Study (follow − up = 20 years) as demonstrated by a decrease in the number of cholecystectomies (*n* = 7831) by more than 20% [7]. In both cohorts, animal protein had no effect on the risk of GSD. Smaller studies were not as conclusive; a prospective cohort of 3070 pregnant women found no significant association between total or subtypes of dietary protein including vegetable protein with GSD or sludge formation [26]. A case–control study on 242 women, 121 of whom were detected to have GSD using ultrasonography, found no difference in total, animal, nonanimal (including vegetable), and animal (including dairy, eggs, etc.) protein intake between GSD cases and controls [19].

Data from large-scale studies regarding total protein in GSD is contradictory. The Women's Health Initiative cohort found total protein to be mildly protective against GSD [8]. Conversely, total protein intake positively correlated with the risk of GSD detected using ultrasonography in an epidemiologic survey of nearly 30,000 individuals, but this correlation was observed only in the male population [11]. The Nurses' Health Study cohort revealed no association between total protein and the risk of cholecystectomy [7], similar to a case–control study in southern Italy which found no association between dietary protein and GSD [9].

Fatty acid content of dietary proteins may partly explain their role in gallstone formation. Dairy products including whole milk, cheese and butter, and many animal-based sources of protein including red meat are high in saturated fatty acids (SFA) [27], while several sources of plant-based protein including soybeans [28], avocado [29], corn [30], and walnuts [31] contain high amounts of polyunsaturated fatty acids (PUFA) and monounsaturated fatty acids (MUFA). Although the exact role of dietary lipids in pathogenesis of GSD is not clear, fatty acids appear to influence the main stages of GSD formation including bile cholesterol supersaturation, bile stasis due to reduced gallbladder motility and cholesterol nucleation [32]. Additionally, vegetable protein appears to decrease biliary cholesterol, biliary lithogenic index and cholesterol precipitation, the earliest step in formation of gallstones, and increase bile acid concentration which is negatively correlated with biliary cholesterol [24].

High total protein consumption above recommended dietary allowance and vegetable protein consumption appear to increase high-density lipoprotein cholesterol (HDL) [33, 34]. Compared to low-protein diets, high-protein diets increase HDL cholesterol. Substitution of meat and dairy protein for carbohydrates in 10 individuals with high cholesterol levels who were on a low-fat, low-cholesterol diet increased the level of HDL cholesterol, while decreasing the levels of total and LDL cholesterols in addition to triglycerides in the serum [35]. Decreased HDL cholesterol levels and increased triglyceride levels in the serum are frequently observed in GSD [36, 37]. HDL cholesterol is involved in bile acid synthesis and bile cholesterol saturation index (CSI) is negatively correlated with HDL cholesterol [38]. CSI is directly associated with plasma triglycerides and negatively associated with HDL cholesterol [39]. Increased CSI decreases cholesterol solubility in bile and consequently increases gallstone formation. This would help explain the protective role of total and vegetable protein in gallstone formation. On the other hand, it appears that total protein concentration in the bile is associated with the presence of bile cholesterol crystals, which may facilitate cholesterol gallstone formation. However, it is not clear if high-protein diet translates to high-protein bile and the observed increase in bile protein could be the result of mucosal inflammation and the subsequent increased mucosal permeability in the gallbladder, which is seen in GSD and allows leakage of plasma proteins into the bile [40]. Furthermore, high protein intake increased cholesterol levels in pigs compared to normal protein intake [24].

Dairy products are high in calcium. Excessive calcium intake may augment gallstone formations. Oral calcium supplementation led to formation of pigment gallstones and calcium bilirubinate sludge in prairie dogs and elevated glycoprotein concentration [41]. Glycoproteins are involved in formation of pigment stones and sludge, and mucin glycoprotein acts as a pro-nucleating agent in GSD by providing a hydrophobic binding site for cholesterol monohydrate to nucleate from supersaturated bile [42]. Detrimental effect of high dairy intake in GSD should be considered when prescribing calcium supplements. Calcium supplements are often prescribed for postmenopausal women to prevent osteoporosis [43], and for pregnant women, two groups that are at increased risk for GSD. Prescribing calcium supplements must be weighed against possible adverse effects. Previous studies point to a possible increase in risk of renal stones [44] and cardiovascular diseases [45] with calcium supplements [46]. A potential increase in risk of GSD as observed in our results should be considered before adding calcium supplements, particularly in the diet of postmenopausal and pregnant women who are at increased risk for GSD. Most studies on the relationship between calcium and GSD have considered calcium regardless of its source, supplements, or dairy products, and therefore, the relationship between dairy products and GSD cannot be definitively attributed to calcium. However, it must be noted that our study only assessed dairy products and we did not evaluate the role of supplements including calcium supplements.

A protein-rich diet may additionally protect against GSD by promoting weight loss, particularly reducing central adiposity as indicated by a significant reduction in waist circumference [33, 47]. Increased body weight is a well-known risk factor for GSD and interestingly, abdominal obesity itself is a risk factor for GSD and cholecystectomy, independent of BMI [48, 49]. Although vegetables and dairy products contain a fair amount of protein, a large part of total protein intake is sourced from animal-based products. The overlap between animal and dairy food sources containing high protein and high saturated fat could in part explain the diminishing benefits of high protein consumption compared to moderate protein consumption. Examples of such include red meat, processed meat including sausage, poultry (e.g. skin-on chicken), whole milk and cheese. As can be seen in Figure 2, the protective effect of vegetable protein (and average dairy protein) is offset by the detrimental effects of animal protein and high amounts of dairy protein. Decreased benefit of total protein intake in GSD appears to be the compound result of animal and dairy protein intake. Also, the potential heterogeneity of animal protein sources should be considered when interpreting the results. It is possible that the effects of white and red meat may be different, but due to the small number of studies available, definitive conclusions cannot be drawn. In interpreting the results of the association of protein intake with GSD risk, the implicit substitution effect of carbohydrates or fats should be considered. Carbohydrate and fat intake, depending on their type and amount, can contribute to the risk of GSD, as previously discussed in separate studies [50, 51]. The conflicting results may in part be explained by the miscellaneous methods employed across studies. Some studies were limited to symptomatic GSD [22, 52], while others enrolled patients on the basis of GSD detection using ultrasound, and thus included both symptomatic and asymptomatic patients symptomatic GSD. Some studies were limited only to males [53] or females [54]. It should be considered that GSD is a lot more likely to occur in females than males, as estrogen hormone is a contributing factor to bile cholesterol secretion and the resultant bile supersaturation [55]. This study attempted to provide a comprehensive view of different types of dietary proteins. We included both males and females, and accounted for sex as a confounding factor in our results. However, no adjustment for age or gender was applied in the present study, and given that the risk of GSD increases with age and is higher in women than in men, this limitation could affect the generalizability of the results. In addition, patients are subject to changing their dietary patterns after receiving a new diagnosis for GSD, making it difficult to compare case–control studies that enroll patients with established GSD with prospective cohorts recruiting initially healthy subjects. Although the FFQ was administered shortly after diagnosis, symptoms preceding the diagnosis of gallstone disease (e.g., intermittent abdominal pain) could have led to dietary changes and influenced the observed associations. To increase accuracy, an experienced dietitian conducted the interviews and completed the questionnaire; however, some degree of bias is inevitable. Even though we accounted for confounders, the likelihood that other unaccounted confounders (including family history of GSD and medications) may have influenced the results cannot be ruled out. Additionally, our collected data may be susceptible to recall bias, as is the case in FFQs. However, in large-scale epidemiological research, FFQ is frequently employed as the primary dietary assessment tool due to its methodological advantages. Compared to open-ended dietary approaches, such as food records, the FFQ facilitates more efficient data analysis, incurs lower costs, and offers greater feasibility for use in extensive population studies. Moreover, by capturing dietary intake over an extended period, the FFQ provides a more representative reflection of habitual dietary patterns than assessments conducted over shorter time frames. Also, as a cross-sectional study, it is not possible to generalize cause-and-effect relationships from our results. The strength of our study was the various types of dietary protein examined, particularly dairy protein, which has seldom been previously explored in studies on dietary protein and GSD.

## 5. Conclusion

The role of dietary protein and its subtypes in development of GSD is contested. Our results offer a protective role for moderate dairy protein, vegetable protein and overall protein consumption, while it appears that animal protein and high dairy protein intake may be a risk factor for development of GSD. Fatty acid content of protein subtypes may in part be responsible for the observed results. It may be advisable for individuals who are at increased risk for GSD to source a higher portion of their dietary protein from vegetables and moderate amounts of low-fat dairy in place of animal resources. Premenopausal and pregnant women should consider the potential effect of high dairy intake on GSD if they are planning to increase calcium intake through dairy consumption, as higher dairy intake may further increase the risk of GSD.

## Figures and Tables

**Table 1:** Baseline general characteristics and dietary intakes of study participants by tertile of total dietary protein intake.

**Tertile of total dietary protein intake (g/day)**				
	**T3 (< 64)** **(** **n** = 177**)**	**T2 (64–82)** **(** **n** = 177**)**	**T1 (82 <)** **(** **n** = 177**)**	*p * **Value**
Cases, *n* (%)	78 (41)	52 (28)	59 (31)	0.006
Men, *n* (%)	49 (24)	67 (33)	86 (43)	< 0.001
Age (years)	54.5 ± 13.5	52.4 ± 13.1	52.1 ± 13	0.188
Alcohol drinker	2	3	7	0.165
Smoker, %	16	26	35	0.015
IPAQ level, %				0.479
1	136 (34)	135 (34)	126 (32)	
2	38 (33)	34 (30)	42 (37)	
3	3 (12)	8 (37)	9 (51)	
Weight, kg	70.6 ± 13.1	73.6 ± 12.4	74.9 ± 14	0.009
Height, cm	163.1 ± 8.2	164.4 ± 8.6	166.6 ± 8.7	< 0.001
BMI, kg/m^2^	26.5 ± 4.3	26.9 ± 4.4	27.4 ± 4.3	0.196
Calorie intake (kcal/day)	1863.8 ± 438.8	2343 ± 400.4	2902.4 ± 539.4	< 0.001
Total protein (g/day)	51.2 ± 10.2	72.5 ± 5.1	100.6 ± 15.9	< 0.001
Dairy protein (g/day)	12.1 ± 6.9	14.1 ± 7.4	17.6 ± 13.6	< 0.001
Vegetable protein (g/day)	30.8 ± 15.9	33.3 ± 12.8	41.7 ± 16.6	< 0.001
Animal protein (g/day)	18.9 ± 15.8	22.3 ± 9.2	30.7 ± 13.8	< 0.001

*Note*: Values are means ± SDs for continuous variables and percentages for categorical variables. ANOVA for quantitative variables and *χ*^2^ test for qualitative variables.

Abbreviations: BMI, body mass index; IPAQ, International Physical Activity Questionnaire.

**Table 2:** Odds and 95% confidence interval for occurrence of the gallstone in each tertile categories of protein intake.

	**Tertiles of dietary protein intake**			***p* trend**
*Total protein*	T1 (< 64)	T2 (64–82)	T3 (82 <)	
No. of cases	78	52	59	0.006
Model 1	Ref	0.5 (0.32–0.78)	0.61 (0.395–0.93)	0.032
Model 2	Ref	0.60 (0.38–0.95)	0.68 (0. 43–1.05)	0.260
Model 3	Ref	0.2 (0.1–0.4)	0.39 (0.23–0.66)	< 0.001
*Dairy protein*	T1 (< 10)	T2 (10–17)	T3 (> 17)	
No. of cases	69	52	68	0.200
Model 1	Ref	0.71 (0.45–1.1)	1.1 (0.65–1.76)	0.007
Model 2	Ref	0.74 (0.48–1.14)	1.24 (0.7–1.81)	0.008
Model 3	Ref	0.76 (0.45–1.21)	1.5 (0.8–1.89)	0.027
*Vegetable protein*	T1 (< 28)	T2 (28–38)	T3 (> 38)	
No. of cases	73	60	56	0.045
Model 1	Ref	0.71 (0.46–1.1)	0.64 (0.42–1)	0.013
Model 2	Ref	0.78 (0.49–1.2)	0.69 (0.45–1.2)	0.044
Model 3	Ref	0.58 (0.36–0.96)	0.55 (0.33–0.93)	0.001
*Animal protein*	T1 (< 29)	T2 (29–40)	T3 (> 40)	
No. of cases	49	69	71	0.029
Model 1	Ref	1.1 (0.7–1.56)	1.34 (0.6–2.3)	0.060
Model 2	Ref	1.22 (0.78–1.85)	1.41 (0.9–2.9)	0.045
Model 3	Ref	1.28 (1.1–2.7)	1.83 (1.1–3.4)	0.038

*Note*: Based on multiple logistic regression models. Model 1: crude. Model 2: adjusted for age and sex. Model 3: additionally adjusted for energy intake, BMI, physical activity, smoking, alcohol.

## Data Availability

The datasets analyzed in the current study are available from the corresponding author on reasonable request.
